# Treatment with recombinant Sirt1 rewires the cardiac lipidome and rescues diabetes-related metabolic cardiomyopathy

**DOI:** 10.1186/s12933-023-02057-2

**Published:** 2023-11-13

**Authors:** Sarah Costantino, Alessandro Mengozzi, Srividya Velagapudi, Shafeeq Ahmed Mohammed, Era Gorica, Alexander Akhmedov, Alessia Mongelli, Nicola Riccardo Pugliese, Stefano Masi, Agostino Virdis, Andreas Hülsmeier, Christian Matthias Matter, Thorsten Hornemann, Giovanni Melina, Frank Ruschitzka, Thomas Felix Luscher, Francesco Paneni

**Affiliations:** 1 Center for Translational and Experimental Cardiology (CTEC), Department of Cardiology, Zurich University Hospital and University of Zurich, Wagistrasse 12, 8952 Schlieren, Switzerland; 2 Department of Cardiology, Zurich University Hospital, Zurich, Switzerland; 3 Department of Clinical and Experimental Medicine, University of Pisa, Pisa, Italy; 4 Health Science Interdisciplinary Center, Sant’Anna School of Advanced Studies, Pisa, Italy; 5 Center for Molecular Cardiology, University of Zurich, Zurich, Switzerland; 6 Institute for Clinical Chemistry, University Hospital and University of Zürich, Zurich, Switzerland; 7 Department of Clinical and Molecular Medicine, Sapienza University of Rome, Rome, Italy; 8 Royal Brompton and Harefield Hospitals and Imperial College, London, UK

**Keywords:** Metabolic cardiomyopathy, Sirt1, Lipidome, Diabetes, Cardiometabolic, Therapy

## Abstract

**Background:**

Metabolic cardiomyopathy (MCM), characterized by intramyocardial lipid accumulation, drives the progression to heart failure with preserved ejection fraction (HFpEF). Although evidence suggests that the mammalian silent information regulator 1 (Sirt1) orchestrates myocardial lipid metabolism, it is unknown whether its exogenous administration could avoid MCM onset. We investigated whether chronic treatment with recombinant Sirt1 (rSirt1) could halt MCM progression.

**Methods:**

*db/db* mice, an established model of MCM, were supplemented with intraperitoneal rSirt1 or vehicle for 4 weeks and compared with their *db/* + heterozygous littermates. At the end of treatment, cardiac function was assessed by cardiac ultrasound and left ventricular samples were collected and processed for molecular analysis. Transcriptional changes were evaluated using a custom PCR array. Lipidomic analysis was performed by mass spectrometry. H9c2 cardiomyocytes exposed to hyperglycaemia and treated with rSirt1 were used as in vitro model of MCM to investigate the ability of rSirt1 to directly target cardiomyocytes and modulate malondialdehyde levels and caspase 3 activity. Myocardial samples from diabetic and nondiabetic patients were analysed to explore Sirt1 expression levels and signaling pathways.

**Results:**

rSirt1 treatment restored cardiac Sirt1 levels and preserved cardiac performance by improving left ventricular ejection fraction, fractional shortening and diastolic function (E/A ratio). In left ventricular samples from rSirt1-treated *db/db* mice, rSirt1 modulated the cardiac lipidome: medium and long-chain triacylglycerols, long-chain triacylglycerols, and triacylglycerols containing only saturated fatty acids were reduced, while those containing docosahexaenoic acid were increased. Mechanistically, several genes involved in lipid trafficking, metabolism and inflammation, such as *Cd36*, *Acox3*, *Pparg*, *Ncoa3*, and *Ppara* were downregulated by rSirt1 both in vitro and in vivo. In humans, reduced cardiac expression levels of Sirt1 were associated with higher intramyocardial triacylglycerols and PPARG-related genes.

**Conclusions:**

In the *db/db* mouse model of MCM, chronic exogenous rSirt1 supplementation rescued cardiac function. This was associated with a modulation of the myocardial lipidome and a downregulation of genes involved in lipid metabolism, trafficking, inflammation, and PPARG signaling. These findings were confirmed in the human diabetic myocardium. Treatments that increase Sirt1 levels may represent a promising strategy to prevent myocardial lipid abnormalities and MCM development.

**Graphical Abstract:**

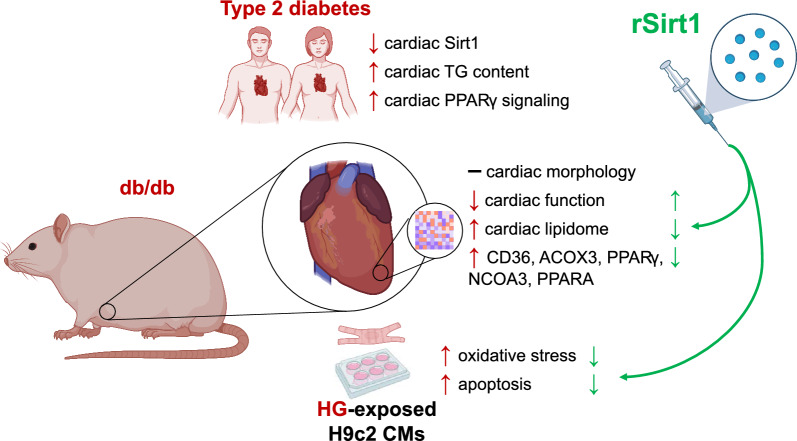

**Supplementary Information:**

The online version contains supplementary material available at 10.1186/s12933-023-02057-2.

## Background

Cardiometabolic diseases, such as obesity and type 2 diabetes (T2D), characterized by dysregulated systemic, organ and tissue-specific metabolic signatures [1, 2], associate with high cardiovascular risk. This affects individuals by increasing mortality and morbidity, as well as the society, overburdening the healthcare systems [3, 4].

Among the constellation of cardiometabolic complications, metabolic cardiomyopathy (MCM) takes the central stage, given its association with incident heart failure and mortality [5]. MCM is defined by cardiac hypertrophy and impaired remodeling, in the absence of coronary artery disease or hypertension [6]. An emerging feature of MCM is represented by disturbed lipid signaling fostering fatty acid (FA) uptake with subsequent intramyocardial lipid accumulation and lipotoxic damage [7, 8]. In turn, FA-driven lipotoxicity damages cardiomyocytes, promoting apoptosis and increasing stiffness, with a consequent decline in contractile function [9]. These events are crucial for the onset and progression of MCM and predispose to structural and functional changes leading to the development of heart failure with preserved ejection fraction (HFpEF) [10, 11].

Sirtuins are key molecules that are upregulated during calorie restriction and have attracted considerable attention over the last two decades due to their deep involvement in the maintenance of metabolic homeostasis [12]. In particular, the mammalian silent information regulator 1 (Sirt1) has been shown to orchestrate pathways involved in the preservation of vascular function [13, 14], insulin sensitivity [15], adipose tissue and liver homeostasis [16]. Sirt1 is involved in FA metabolism [17] and mitochondrial function [13], both of which are compromised in MCM and HFpEF [18]. Furthermore, the restoration of Sirt1 activity has been proposed as the mechanism underlying the beneficial effects of SGLT-2 inhibitors (SGLT-2i) on the cardiovascular system [11, 19, 20].

However, although Sirt1-targeting approaches have been proposed to rescue cardiac function in the context of MCM [21], evidence is scarce. No studies have previously explored the direct supplementation of Sirt1 rather than its pharmacological activation on cardiac function. We aimed to investigate whether preserving Sirt1 levels via exogenous supplementation can affect the MCM phenotype. Thus, we designed a preclinical study in which a mouse model of MCM was administered chronically a mouse recombinant Sirt1 (rSirt1), investigating its effect on cardiac function, myocardial lipid signature and the related transcriptional programmes. In a translational approach, we confirmed our findings in an in vitro model of metabolic stress and in myocardial specimens from patients with and without T2D.

## Methods

### MCM mouse model and study design

Male C57BKS/Leprdb (*db/db*) mice and *db/* + heterozygous littermates purchased from The Jackson Laboratory were used for the in vivo study as a mouse model of MCM. Mouse rSirt1 (CSB-EP846058MO, Cusabio) administered by intraperitoneal injection (0.3 mg/Kg every other day over a 4-week period) was used to investigate the impact of Sirt1 restoration on the MCM phenotype (Additional file 1: Figure S1). Three groups of 16-week-old mice were studied: 1) *db/db* mice treated with rSirt1 (n = 12); 2) *db/db* mice treated with vehicle [n = 13); 3] heterozygous *db/* + (n = 11). All mice were housed in temperature-controlled cages (20 °C–22 °C) and maintained on a 12/12-h light/dark cycle. Except where indicated, mice had access to normal chow and water ad libitum. Animal experiments were performed in accordance with our institutional guidelines and were approved by the veterinary authorities of the Canton of Zürich (ZH033/2021).

### In vivo assessment of cardiac function by echocardiography

Cardiac function was assessed in mice by transthoracic echocardiography by using a Vevo 3100 (VisualSonics, Toronto, Canada) high-resolution imaging system equipped with a 22–55 MHz (MX400) linear array transducer [8]. Mice were anesthetised with isoflurane (2–5%)/oxygen (1L/min) and placed on a temperature-controlled operating table to maintain the rectal temperature at 37 °C. Measurements were obtained from grayscale M-mode and B-mode images, at the midpapillary level in the parasternal short-axis view. Conventional measures of the left ventricle (LV) included: end-diastolic internal diameter (LVIDd), end-systolic internal diameter (LVIDs), anterior and posterior wall thicknesses, end-systolic (ESV) and end-diastolic volumes (EDV), LV mass, fractional shortening (FS), and ejection fraction (EF). FS was calculated as [(LVIDd–LVIDs)/LVIDd] × 100, while EF was assessed using the following formula: [(EDV–ESV)/EDV] × 100. The trans-mitral inflow pattern was measured in the apical 4-chamber to assess early (E) and atrial (A) peak filling rates. Tissue Doppler Imaging (TDI) of the posterior LV wall in the short-axis view was used to assess the radial axis, as previously reported [22]. The TDI waveform was used to measure the following parameters: isovolumic relaxation time (IVRT), E’ and A’ myocardial diastolic velocities and isovolumic contraction time (IVCT) and aortic ejection time (AET). Myocardial performance index (MPI), an overall indicator of systolic and diastolic dysfunction, was calculated using the following formula: (IVRT + IVCT)/AET. All images were analysed using the Visual Sonics software. The investigators performing and reading the echocardiograms were blinded to the treatment allocation.

## Measurements of Sirt1 protein levels

The levels of Sirt1 were measured in murine left ventricular samples by a commercially available ELISA kit (ab206983, Abcam, Cambridge, United Kingdom), according to the manufacturer’s protocol.

### Lipidomic analysis

Lipid extraction was performed on murine left ventricular samples as previously described [8, 23], with some modifications. To 20 µl of the sample, 1 ml of a mixture of methanol: MTBE: chloroform (MMC) 1.33:1:1 (v/v/v) was added. The MMC mixture was supplemented with the SPLASH mix internal standard and additional internal standards: d7-sphinganine (SPH d18:0), d7-sphingosine (SPH d18:1), dihydroceramide (Cer d18:0/12:0), ceramide (Cer d18:1/12:0), deoxydihydroceramide (Cer m18:0 12:0) deoxyceramide (Cer m18:1 12:0) and glucosylceramides (GlcCer d18:1 18:1 (d5)) (Avanti Polar Lipids). After brief vortexing, the samples were continuously mixed in a Thermomixer (Eppendorf) at 25 °C (950 rpm, 30 min). Protein precipitation was obtained after centrifugation for 10 min, 16,000 g, 25 °C. The single-phase supernatant was collected, dried under N2 and stored at -20 °C until analysis. Prior to analysis, the dried lipids were redissolved in 100µL MeOH. Liquid chromatography was performed as previously described [24], with some modifications. Lipids were separated using C30 reverse-phase chromatography. Transcend TLX eluting pump (Thermo Scientific) was used with the following mobile phases: A) Acetonitrile:Water (6:4) with 10 mM ammonium acetate and 0.1% formic acid and B) Isopropanol: Acetonitrile (9:1) with 10 mM ammonium acetate and 0.1% formic acid. The C30 Accucore LC column (Thermo Scientific) with the dimensions of 150 mm*2.1 mm*2.6 µm (length*internal diameter*particle diameter) was used. The following gradient was used with a flow rate of 0.26 ml/min; 0.0–0.5 min (isocratic 30%B), 0.5–2 min (ramp 30–43% B), 2.10–12.0 min (ramp 43–55%B),12.0–18.0 min (ramp 65–85%),18.0–20.0 min (ramp 85%-100%B), 20–35 min (isocratic 100%B), 35–35.5 min (ramp 100–30% B) and 35.5–40 min (isocratic 30%B). Liquid chromatography was coupled to a hybrid quadrupole-orbitrap mass spectrometer (Q-Exactive, Thermo Scientific). Data-dependent acquisition with positive and negative polarity switching was used. A full scan was used scanning from 220 to 3000 m/z at a resolution of 70,000 and AGC Target 3e6, while data-dependent scans (top10) were acquired using normalized collision energies (NCE) of 25, 30 and a resolution of 17,500 and AGC target of 1e5. Lipid species identification was achieved using four criteria: (1) high accuracy and resolution with an accuracy within m/z of 5 ppm shift from the predicted mass and a resolving power of 70,000 at 200 m/z; (2) isotopic pattern matching the expected isotopic distribution; (3) comparison of the expected retention time with an in-house database and 4) identification of species-specific fragments m/z in positive and negative mode.

### Real time PCR

Total RNA was extracted from mouse and human myocardium using TRIzol Reagent (Invitrogen, Carlsbad, CA) according to the manufacturer’s recommendations. Prior to extraction, mouse and human myocardial samples were lysed by using a Precellys homogenizer. Total cellular RNA was converted to cDNA using Moloney murine leukaemia virus reverse transcriptase and random hexamers (Amersham Bioscience, Piscataway, USA) in a final volume of 33 μl, using 1 μg of cDNA according to the manufacturer’s recommendations. Real-time PCR was performed using the SYBR Select Master Mix (Applied biosystems, Thermo Fischer Scientific, Zug, Switzerland) together with the gene-specific primers on a Quant Studio 5 and 7 cyclers (Life Technologies, Thermo Fischer Scientific, Zug, Switzerland) according to the manufacturer’s instructions. *GAPDH* or *TBP* were used as endogenous controls to normalise the RNA concentration. The amplification programme consisted of 1 cycle at 95 °C for 10 min, followed by 40 cycles with a denaturing phase at 95 °C for 30 s and an annealing and elongation phase of 1 min at 60 °C. Melting curve analysis was performed after amplification to verify the accuracy of the amplicon. Differences in Ct values between the test gene and endogenous controls (*GAPDH* and *TBP*, ΔCt) were calculated and used for statistical analysis.

### Experiments in cultured cardiomyocytes

H9c2 cardiomyoblasts (Lonza, Bettlach, Switzerland) were cultured using M199 complete media [M199 media supplemented with 10% fetal bovine serum, 1% penicillin and streptomycin, (Sigma-Aldrich, Steinheim, Germany)] and treated with either normal glucose (5 nM) or high glucose (25 nM) for 72 h, in the presence or in the absence of rSirt1 (10 nmol/L) or vehicle. After treatment, cells were lysed in ice-cold lysis buffer containing 50 mM Tris–HCl, pH 7.4, 100 mM NaF, 15 mM Na_4_P_2_O_7_, 1 mM Na_3_VO_4_, 1% Triton X-100, and 1 mM phenylmethylsulfonyl fluoride. Lysates were centrifuged at 10,000 g to remove insoluble material.

### Measurements of malondialdehyde levels

The levels of malondialdehyde (MDA), a marker of peroxynitrite-mediated oxidative damage, were measured in cell lysates by a commercially available ELISA kit (ab238537, Abcam, Cambridge, United Kingdom) according to the manufacturer’s protocol.

### Caspase-3 activity assay

Caspase-3 activity was assessed using a colourimetric activity assay (ab39401, Abcam, Cambridge, United Kingdom) according to the manufacturer’s protocol.

### Study population

From March 2016 to February 2018, diabetic patients (n = 9) and nondiabetic age-matched controls (n = 9) were consecutively recruited at the Department of Cardiac Surgery, Sant’Andrea Hospital, Rome, Italy. Patients and controls were selected among those undergoing cardiopulmonary bypass for surgical valve replacement or coronary artery bypass grafting (CABG). The following patients were excluded: (i) overt signs of cardiomyopathy (i.e. clinical signs of heart failure, left ventricular ejection fraction < 50%, left atrial dilatation (> 40 mm), systolic pulmonary artery pressure > 40 mm Hg, or brain natriuretic peptide level > 100 ng/L); (iii) history of atrial fibrillation/atrial flutter; (iv) stenosis > 50% of the right coronary artery. Patients were also excluded when the amount of tissue was too small to assess lipid content and molecular analyses. T2D diagnosis was made according to current recommendations [25]. Clinical and echocardiographic data (including TDI) were obtained at admission. Right atrial tissue was collected during cannulation of the right atrium in preparation for cardiopulmonary bypass. Samples of the appendage were immediately prepared for molecular studies, and the remaining tissue was frozen in liquid nitrogen. The study protocol was approved by the Local Ethics Committee, and in accordance with institutional guidelines. All participants were aware of the investigational nature of the study and gave written consent for their participation.

### Triacylglycerol content in heart tissue

Total triacylglycerol (TAG) content was determined in human myocardium homogenates using a commercially available kit (Triglyceride Colorimetric Assay Kit, n 10,010,303, Cayman Chemicals) following the manufacturer’s instructions.

### Statistical analysis

Data are presented as median [IQR]. Group-wise comparisons were conducted using the Mann–Whitney U test (two groups), the Kruskal–Wallis test with Dunn post hoc test for continuous variables (multiple groups) and the Fischer’s exact test for categorical variables. When multiple measurements were performed on the same sample, comparisons were corrected for multiple testing by the two stage step-up Benjamini, Krieger and Yekutieli false discovery rate method at an alpha level of 0.05. Correlations between variables were assessed by Spearman’s test. A value of P < 0.05 was considered statistically significant. All analyses were performed using GraphPad Prism (GraphPad Software, version 9.04).

## Results

### rSirt1 treatment preserves cardiac function in db/db mice

**Fig. 1:**
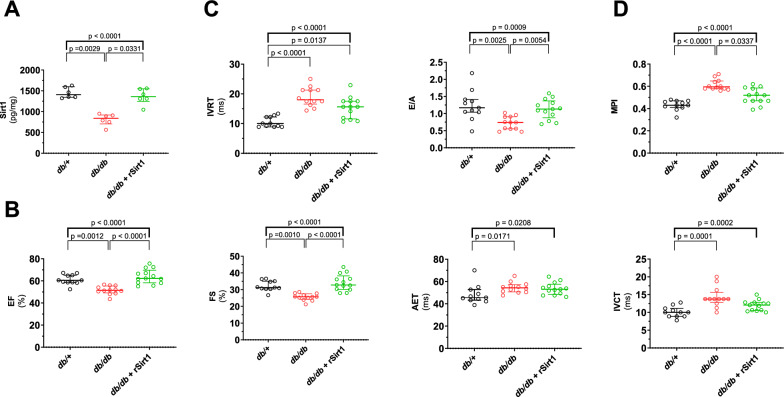
rSirt1 treatment rescues cardiac function in a mouse model of MCM. **A** Myocardial levels of rSirt1 in the three experimental groups. **B** Indices of systolic function (ejection fraction, fractional shortening, aortic ejection time and isovolumic contraction time) across the three experimental groups. **C** Diastolic function assessed *as* isovolumic relaxation time and E/A in the three experimental groups. **D** Myocardial performance index in the three experimental groups. Data are presented as box plots showing median [IQR] and compared by the Kruskal–Wallis test (upper bold bar) with Dunn post hoc test. *p < 0.05, **p < 0.01. *AET* aortic ejection time, *EF* ejection fraction, *FS* fractional shortening *IVCT* isovolumic contraction time, *IVRT* isovolumic relaxation time *MPI* Myocardial performance index *rSirt1* recombinant Sirt1

In the *db/db* mice, myocardial levels of Sirt1 were reduced (Fig. 1A), and this associated with impaired cardiac function and structure. The cardiac morphology of 16-week-old *db/db* mice showed a small LV cavity described by smaller LVIDd, LVIDs, reduced EDV and ESV with normal LV mass (Additional file 1: Figure S2A). Stroke volume and heart rate were all reduced, resulting in a significant reduction of cardiac output (Additional file 1: Figure S2B). Systolic function was impaired in diabetic mice as assessed by ejection fraction, FS, AET and IVCT (Fig. 1B). Diastolic performance was also affected, as shown by a prolonged IVRT and reduced E’/A’ ratio (Tissue Doppler analysis) (Fig. 1C). PI, a reliable indicator of systo-diastolic cardiac performance, was almost doubled in diabetic mice, confirming an impairment of cardiac function in our MCM model (Fig. 1D). In the *db/db* mice, rSirt1 supplementation over a period of 4 weeks was associated with a restoration of Sirt1 levels (Fig. 1A) and resulted in a significant improvement of both systolic and diastolic cardiac function, with a less pronounced effect on LV remodeling (Fig. 1B–D, Additional file 1: Figure S2). Regarding cardiac function, stroke volume was comparable to that observed in control mice. No difference in heart rate was observed, with only a partial preservation of the cardiac output (Additional file 1: Figure S2B). EF and FS were superimposable to controls, while no difference was observed in AET and IVCT (Fig. 1B). Diastolic function was completely preserved regarding E/A, with only a mild effect on IVRT observed (25% reduction, still 1.5 fold higher than control mice; Fig. 1C). The sum of these improvements attenuated MPI derangement by 20%, to a level still higher than controls (Fig. 1D).

### rSirt1 rewires the cardiac lipidome in MCM

**Fig. 2:**
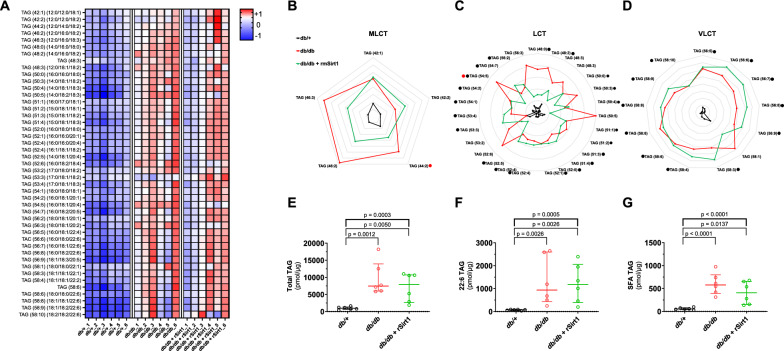
Cardiac lipidomic signature in MCM is modulated by rSirt1 treatment. **A** Heat map showing levels of different lipid species across the three experimental groups (n = 6 for each group). **B-D** Radar plots describing Medium and long-chain triacylglycerols, long-chain triacylglycerols, and very long-chain triacylglycerols in the three experimental groups. Red dots: statistically significant difference between *db/db* mice treated with rSirt1 and *db/db* mice. Black dots: statistically significant difference between *db/db* mice treated with rSirt1 and *db/* + mice; **E–G** Total triacylglycerols content, total 22:6- containing triacylglycerols content, total triacylglycerols containing only saturated fatty acids content in the three experimental groups. Data are presented as median and internal normalised within each species to provide a standardised measurement **A-D;** in radar plots, data are expressed in log_10_ changes and each grey line represent a 0.2 fold change) or as box plots **E–G** showing median [IQR] and compared by the Kruskal–Wallis test (upper bold bar) and corrected for multiple testing by the two stage step-up Benjamini, Krieger and Yekutieli false discovery rate method at an alpha level of 0.05. *p < 0.05, **p < 0.01. Full data are reported in Supplementary Table 1. *LCT* Long-chain triacylglycerols *MLCT* medium and long-chain triacylglycerols, *rSirt1* recombinant Sirt1, *SFA* saturated fatty acid,* TAG* triacylglycerols *VLCT* very long-chain triacylglycerols

In LV samples isolated from 16-week-old *db/db* mice, lipidomic analysis showed an accumulation of different TAGs (Fig. 2A, Additional file 1: Table S1). Medium and long-chain TAGs (MLCT), long-chain TAGs (LCT), and very long-chain TAGs (VLCT) were all increased (Fig. 2B-D), suggesting an upregulation of fatty acid metabolism with both increased FA uptake and de novo synthesis. Total lipid content was not affected by rSirt1 treatment (Fig. 2E). However, intramyocardial levels of several lipid species were significantly different in the 16-week-old *db/db* mice treated with rSirt1 compared to vehicle. MLCT and LCT were reduced, whereas rSirt1 did not appear to influence VLCT levels (Fig. 2B-D). TAGs containing at least one 16:0 FA were also unaffected by rSirt1 treatment, whereas those containing docosahexaenoic acid (22:6) showed a non-significant (p = 0.2901) increase in the treated mice compared to the *db/db*. TAGs containing only saturated fatty acids (SFAs) showed a non-significant trend towards reduction when comparing treated mice to the *db*/*db* ones (p = 0.1354) (Figs. 2E-G).

### Transcriptional mechanisms affected by exogenous Sirt1 supplementation

**Fig. 3:**
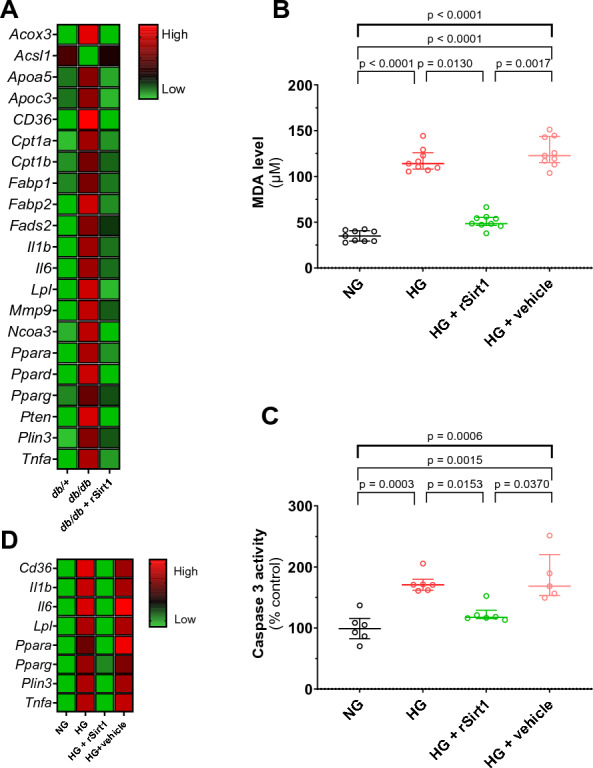
Mechanisms underpinning rSirt1-induced preservation of cardiac function in MCM. **A** Heat map showing differential mean relative expression levels of genes involved in lipid metabolism, trafficking and inflammation in the three experimental groups (n = 4 for each group). **B–C** In vitro assays showing levels of oxidative stress and apoptosis in H9c2 cardiomyocytes exposed to normal glucose (black dots and plots; n = 6), high glucose (red dots and plots; n = 6), high glucose and rSirt1 (green dots and plots; n = 6), high glucose and vehicle (pink dots and plots; n = 6). Data are presented as box plots showing median [IQR] and compared by the Kruskal–Wallis test (upper bold bar) with Dunn post hoc test. *p < 0.05, **p < 0.01. **D** Heat map showing differential mean relative expression levels of genes involved in lipid metabolism, trafficking and inflammation in the four experimental groups (n = 5 for each group). *HG* high glucose *MDA* malondialdehyde; *NG* normal glucose; *rSirt1* recombinant Sirt1

Unbiased gene expression profiling showed upregulation of genes related to MCM and involved in fatty acid transport (*Apoa5*, *Apoc3*, *Cd36*, *Fabp1*, *Fabp2*, *Lpl*, *Plin3*), metabolism (*Acox3*, *Cpt1a*, *Cpt1b*, *Fads2*, *Noca3*, *Ppara*, *Ppard*, *Pparg*, *Pten*) as well as inflammatory response related (*Ncoa3*, *Mmp9, Il1b, Il6, Tnfa*) [26, 27] in LV samples from 16-week-old *db/db* mice, with the sole exception of *Acsl1* which was downregulated (Fig. 3A, Additional file 1: Table S2). Treatment with rSirt1 prevented such transcriptional alterations. Specifically, *Cd36*, *Acox3*, *Pparg*, *Ncoa3, Ppara* genes were the most downregulated by rSirt1 treatment (Fig. 3A, Additional file 1: Table S2).

### rSirt1 treatment mitigates oxidative stress and reduces apoptosis in cardiomyocytes exposed to hyperglycaemia

We designed our in vitro experiment to confirm that rSirt1 treatment directly affects cardiomyocytes and to appraise whether it can protect against signaling pathways underpinning metabolic damage. We exposed H9c2 cells to ambient hyperglycaemia, a well-established model mimicking T2D features in vitro. We observed that high glucose concentration increased MDA, a marker of oxidative stress, and caspase-3 activity, used to assess apoptosis. rSirt1 treatment prevented metabolic injury by mitigating oxidative stress and apoptosis to a level almost superimposable to normal glucose conditions (Fig. 3B-C). Moreover, we also found that rSirt1 treatment was able to rescue HG-induced deregulation of genes implicated in liptoxic damage and cardiomyocyte inflammation such as as *Cd36*, *Il1b*, *Il6*, *Lpl*, *Ppara*, *Pparg*, *Plin3*, *Tnfa* (Fig. 3D, Additional file 1: Table S3).

### Decreased Sirt1 signaling in myocardial samples from T2D patients

**Fig. 4:**
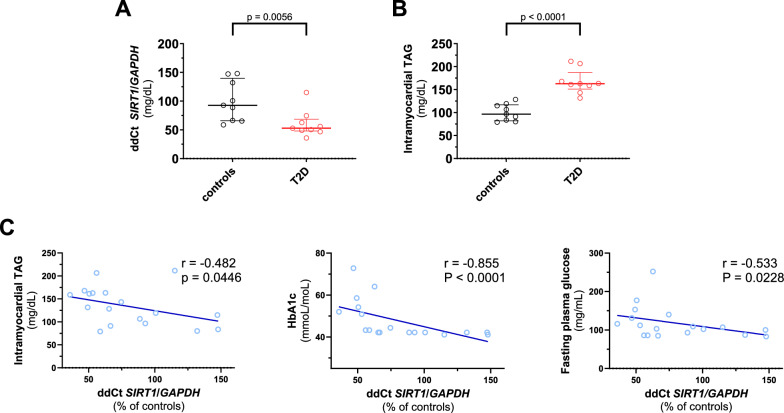
Sirt1 signalling is related to MCM features in human myocardial samples from diabetic and nondiabetic patients. **A** Expression levels of Sirt1 assessed by real-time PCR in myocardial samples from diabetic (n = 9) and nondiabetic (n = 9) patients. **B** Intramyocardial triacylglycerol levels in myocardial samples from diabetic (n = 9) and nondiabetic (n = 9) patients. **C** Scatterplot showing the correlation of myocardial Sirt1 levels with myocardial triacylglycerols, plasma HbA1c and fasting plasma glucose in the study population (n = 18). Data are presented as box plots showing median [IQR] and compared by the Mann–Whitney U test. Correlations between variables were assessed by Spearman’s test. *p < 0.05, **p < 0.01. *T2D* type 2 diabetes *TAG* triacylglycerols

**Fig. 5:**
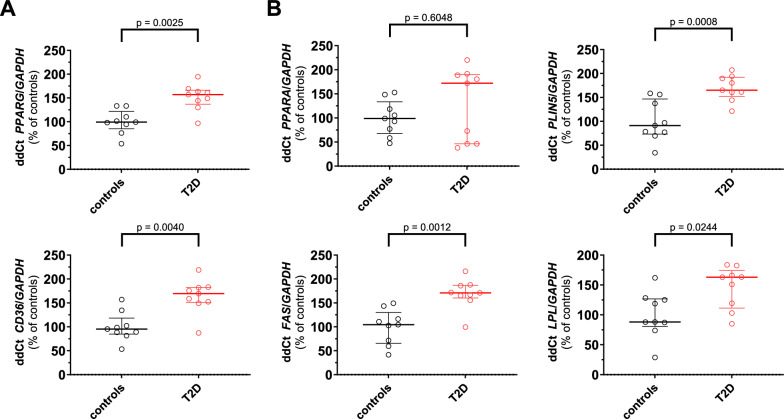
*PPARG* and *PPARG*-related genes in human myocardial specimens. Expression levels of PPARG and PPARG-downstream genes assessed by real-time PCR in myocardial samples from diabetic (n = 9) and nondiabetic (n = 9) patients. Data are presented as violin plots showing median [IQR] and compared by the Mann–Whitney U test. Correlations between variables were assessed by Spearman’s test. *p < 0.05, **p < 0.01. *CD36* cluster of differentiation 36, *FAS* fas cell Surface death receptor, *GAPDH* Glyceraldehyde-3-phosphate dehydrogenase. *LPL* lipoprotein lipase *PLIN5* perilipin 5, *PPARA* peroxisome proliferator-activated receptor alpha, *PPARG* peroxisome proliferator-activated receptor gamma, *T2D* type 2 diabetes

To translate our experimental findings to the human setting, we examined Sirt1 signaling in right atrial samples from T2D patients and age-matched non-T2D individuals. T2D patients showed higher fasting plasma glucose levels, HbA1c and a trend (p = 0.079) towards a reduction in E/A (Additional file 1: Table S4). Consistent with our in vivo experiments, cardiac *SIRT1* expression levels were reduced in T2D patients, whereas intramyocardial TAG levels were increased (Fig. 4A-B). *SIRT1* levels were negatively associated with intramyocardial TAG, a hallmark of MCM, as well as with fasting plasma glucose and HbA1c (Fig. 4C). Given the well-established molecular link between Sirt1 and PPARG signaling [28] and the detrimental role played by PPARG in the context of MCM [8], we examined *PPARG* and *PPARG*-dependent genes *LPL*, *PLIN*, *CD36*, *FAS* and *PPARA* in cardiac specimens from diabetics and controls (Fig. 5). Except for *PPARA*, all *PPARG*-dependent transcripts were upregulated in the diabetic myocardium.

## Discussion

In our study, we show for the first time that: (1) a 4-week supplementation with rSirt1 preserves cardiac function in a murine model of MCM; (2) this effect is associated with a modulation of the cardiac lipid signature in *db/db* mice treated with rSirt1; (3) rSirt1 modulation is associated with the reversal of transcriptional programmes involved in FA metabolism and protects cardiomyocytes against lipotoxic injury; (4) in human myocardial specimens, reduced Sirt1 expression levels are associated with an increase in intramyocardial TAG content and upregulation of PPARG signaling. Our translational findings demonstrate the central role of Sirt1 in maintaining cardiac homeostasis and modulating the myocardial lipidome in the context of diabetes. Restoration of Sirt1 levels by exogenous administration protected against the MCM phenotype and lipotoxic injury, suggesting that rSirt1 boosting may prove a promising strategy to prevent myocardial steatosis and dysfunction in the setting of cardiometabolic disease.

MCM is a major cardiometabolic comorbidity characterized by structural and functional cardiac alterations [5, 29] and represents a crucial step toward progression to HFpEF [30–32]. We report here that chronic treatment with rSirt1 is able to prevent features of MCM and preserve cardiac function. Several studies have highlighted the central role of Sirt1 in maintaining cardiometabolic health [13]. Concerning myocardial homeostasis, in vivo observations have shown that Sirt1 is downregulated in failing hearts [33], and its loss of function by gene deletion leads to the development of a dilated cardiomyopathy phenotype [34]. No previous study has investigated the effect of exogenous supplementation of Sirt1 on MCM. Some observations showed that streptozotocin Wistar rats with type 1 diabetic cardiomyopathy treated with green tea catechins or curcumin, both indirect inducers of Sirt1 activity, had restoration of cardiomyocyte contractility, mitochondrial function and energy availability [35, 36]. In vitro studies have also shown that Sirt1 activation restored cardiomyocyte function by targeting mitochondrial pathways [37]. In our study, we demonstrate that chronic treatment with rSirt1 is able to preserve cardiac function in vivo in a mouse model of MCM. Furthermore, our data are consistent with what has been shown in a more advanced disease model (*db/db* mouse treated with angiotensin II for 4 weeks), in which calorie restriction preserved Sirt1/PGC-1α activity and eventually improved cardiac function [38]. In our study, rSirt1 affected cardiac function rather than structure. Previous observations have shown that resveratrol—a Sirt1-activating compound [39]—improved cardiac geometry in an experimental model of type 1 diabetes [40]. However, cardiac remodelling in the *db/db* mouse reflects the MCM more accurately, as it combines both obesity and T2D. In MCM, the remodeling is also less severe; we would have possibly needed a more prolonged treatment and observation to detect an effect on cardiac geometry [29]. Our findings are consistent with observations on Sirt1 activators, *e.g.* resveratrol [41] or calorie restriction [38], where cardiac function recovery occurs earlier than cardiac structure. However, we cannot exclude the possibility that a longer treatment with rSirt1 might also improve LV morphology.

Intramyocardial lipid accumulation constitutes a hallmark of MCM, as diabetes and obesity disrupt metabolism at both systemic and tissue levels [8]. Increased FA uptake and metabolism lead to overloading of metabolic pathways, resulting in mitochondrial dysfunction, low-grade inflammation and, ultimately, heart failure [42]. We found that supplementation with Sirt1 modulates the cardiac lipid signature but not total TAG levels. Previous studies in patients with T2D showed that lipid quality, not quantity, is altered in the MCM [43], while others demonstrated that prolonged calorie restriction slightly reduces lipid content [44]. Whether a longer treatment duration could have led to quantitative changes in lipid species remains to be determined. However, in qualitative terms, rSirt1 treatment showed a trend towards the reduction of TAGs containing only SFAs and the increase of TAGs containing docosahexaenoic acid [45], the latter being associated with cardioprotective effects by direct modulation of autophagy in cardiomyocytes [46]. Furthermore, the predominant effect on MLCT suggests an important modulation of de novo FA synthesis [47]. Our findings demonstrate for the first time a critical involvement of Sirt1 in myocardial lipid metabolism and trafficking, supporting the notion that rSirt1 promotes beneficial effects on the myocardial lipid pattern. However, further studies are needed to elucidate the impact of lipidomic alterations on myocardial function in this setting.

To identify the putative mechanisms responsible for these changes, we examined the myocardial transcriptional programmes regulated by Sirt1. At the myocardial level, we found an upregulation of several genes involved in FA metabolism and trafficking as well as inflammatory response in our *db/db* mice. Of note, we found that rSirt1 was able to blunt the expression of pivotal inflammatory citokines, namely IL6, IL1β and TNFα, which have shown an important role in advesre cardiac remodeling and progression to heart failure in the setting of MCM [29]. Our results are consistent with those observed by other groups [48, 49]. In our mice, we found that *Acsl1* was downregulated in LV myocardial samples. This may appear partially at odds with previous work showing that *Acsl1* knockdown preserves cardiac function [50] while its overexpression is detrimental to the heart [51]. *Acsl1* downregulation in the untreated *db*/*db* mice could thus be a compensatory mechanism preceding the failing heart stage. Accordingly, rSirt1 treatment did not lead to *Acsl1* overexpression but maintained its expression at the physiological levels observed in healthy hearts. rSirt1 treatment prevented diabetes-related deregulation of several genes implicated in lipotoxic transcriptional programs. Nevertheless, the fact that rSirt1 treatment prevented deleterious changes in transcriptional pathways involved in metabolic dysregulation, as well as in ageing and cancer-related pro-inflammatory pathways, supports the notion that targeting Sirt1 may help prevent a variety of comorbidities at the crossroads of metabolism, ageing and inflammation.

To investigate whether our findings hold true in patients with diabetes, we explored Sirt1 signaling and lipogenic transcriptional programs in myocardial specimens from diabetic patients and controls. In line with our results in mice, Sirt1 was downregulated in the human diabetic myocardium and negatively correlated with total intramyocardial TAG content, a main feature of MCM [8, 29]. Sirt1 also showed a negative correlation with plasma glucose levels and HbA1c, established biomarkers of glucometabolic derangement [52]. Furthermore, we report that Sirt1 is tightly connected with PPARG signaling in the human diabetic myocardium, as shown by the negative correlation between Sirt1 and PPARG gene expression. Previous evidence has shown that Sirt1 negatively modulates PPARG by deacetylation, thus promoting beneficial metabolic effects in several cell types [28, 53]. On the other hand, PPARG was found to directly interact with Sirt1, thus inhibiting its activity and promoting the onset of a negative loop feedback, leading to accelerated cell and tissue senescence [54]. Taken together, our findings imply that perturbations of Sirt1/PPARG signaling are a pivotal step in fostering maladaptive metabolic changes in the heart and MCM development.

Our findings are particularly relevant from a therapeutic standpoint. Calorie restriction is a potent Sirt1 activator [55], and some established drugs, such as metformin and atorvastatin, upregulated *Ampk1* and *Sirt1* in H9c2 cardiomyocytes and heart tissue [56]. Restoration of Sirt1 activation has also been proposed by some authors as one of the mechanisms involved in the beneficial effect of SGLT-2i [19]. Another observation shows how an NAD + -rich diet is cardioprotective, reducing the incidence of HFpEF, which is the link between metabolic dysfunction and cardiovascular risk [2]. Finally, the recent resurgence of interest in the pharmacological Sirt1 supplementation or the restoration of its activity as an anti-ageing strategy [12] could lead to a potentially rapid clinical translation, as several Sirt1 activators are currently undergoing phase II and phase III trials, with the expectation of rapid approval by the major pharmaceutical regulatory agencies.

Nevertheless, our study has some limitations that need to be acknowledged. Firstly, we have not directly shown whether the beneficial effect of Sirt1 supplementation holds true in the human MCM setting. However, the translation of our findings and the beneficial effects already demonstrated for Sirt1 in the context of cardiometabolic disease [13, 57] strongly support the notion that Sirt1 modulation in the human diabetic myocardium could rescue functional changes. Secondly, we focused on Sirt1-dependent transcriptional regulation of lipid genes but did not investigate the possible influence of post-transcriptional regulation in our context. However, most available evidence supports a transcriptional regulation of PPARG-dependent genes by Sirt1 [28, 53]. Thirdly, the TAG measurement and lipidomics data explored in mice were not repeated in the H9c2 cardiomyocytes exposed to HG. However, we believe that a lipidomic analysis in cultured cardiomyocytes exposed to HG would not fully mirror the lipotoxic changes occurring in the diabetic cardiomyopathy phenotype (where hyperglycemia and insulin resistance exert a synergic effect) and would therefore be less informative than the assessment of cardiac lipidome in mice. Fourthly, we have not measured Sirt1 protein levels after rSirt1 treatment in our in vitro model. However, we could confirm that rSirt1 treatment was able to induce similar transcriptional changes in cultured cardiomyocytes, indicating a relevant biological effect of rSirt in this setting. Fifthly, cardiomyocyte apoptosis was assessed by caspase-3 and the use of additional survival assays would have strengthened our evidence of rSirt1 ability to preserve cardiomyocyte viability. Finally, we did not study the circulating or tissue levels of Sirt1 in organs other than the heart (*i.e.*, liver, skeletal muscle and adipose tissue). An effect of rSirt1 on these metabolic organs could have indirectly contributed to the observed improvement of cardiac performance [58]. A systemic effect of rSirt1 could have also contributed to explain the heterogeneous effects on cardiac lipidome remodelling. Nevertheless, the restoration of Sirt1 cardiac levels and our in vitro experiments suggest that the beneficial effect of rSirt1 is mediated, at least in part, by a direct action on the heart.

## Conclusions

In conclusion, our study shows an unprecedented role of rSirt1 in modulating the cardiac lipidome in the setting of MCM and preventing myocardial dysfunction. Collectively, our findings support the beneficial association of Sirt1 levels in humans in light of numerous non-pharmacological and pharmacological strategies such as calorie restriction, metformin, SGLT-2i and statins. Furthermore, they highlight the need for pharmacological Sirt1 activators to be exploited in the human setting in order to prevent cardiometabolic disorders.

### Supplementary Information

**Additional file1: Figure S1.** Schematic study protocol. At 12 weeks, db/db mice were administered recombinant Sirt1 (rSirt1) by intraperitoneal injection, 0.3 mg/Kg every other day. At 16 weeks, mice cardiac function and structure were evaluated, and then mice were sacrificed and harvested for molecular analyses. rSirt1 recombinant Sirt1. **Figure S2**. rSirt1 treatment effect on cardiac geometry and physiology.** A** Left ventricular end-diastolic and end-systolic internal diameters, volumes and mass of the three experimental groups: db/+ mice (black dots and plots; n=11); db/db mice (red dots and plots; n=12); db/db mice after 4-week treatment with rSirt1 (green dots and plots; n=13). **B** Stroke volume, heart rate and cardiac output of the three experimental groups. Data are presented as violin plots showing median [IQR] and compared by the Kruskal-Wallis test (upper bold bar) with Dunn post hoc test. *p<0.05, **p<0.01. EDV end-diastolic volume, ESV end-systolic volume, LVIDd left ventricular end-diastolic internal diameter, LVIDs left ventricular end-systolic internal diameter; rSirt1: recombinant Sirt1. **Table S1**. Cardiac lipidomics analysis performed in the three experimental groups (n=6 for each group). Data are presented as median [IQR] and compared by the Kruskal-Wallis test and corrected for multiple testing by the two stage step-up Benjamini, Krieger and Yekutieli false discovery rate method at an alpha level of 0.05. TAG triacylglycerol. **Table S2**. Expression level data of genes involved in lipid metabolism, trafficking and inflammation in the three experimental groups (n=4 for each group). Data are presented as mean±SD and compared by the two-way ANOVA and corrected for multiple testing by the two stage step-up Benjamini, Krieger and Yekutieli false discovery rate method at an alpha level of 0.05. Acox3 Acyl-CoA Oxidase 3, Acsl1 Acyl-CoA Synthetase Long Chain Family Member 1, Apoa5 Apolipoprotein A5, Apoc3 Apolipoprotein C3, Cd36 Cluster of differentiation 36, Cpt1a Carnitine Palmitoyltransferase 1A, Cpt1b Carnitine Palmitoyltransferase 1B, Fabp1 Fatty Acid Binding Protein 1, Fabp2 Fatty Acid Binding Protein 2, Fads2 Fatty Acid Desaturase 2, Lpl Lipoprotein Lipase, Mmp9 Matrix Metallopeptidase 9, Ncoa3 Nuclear Receptor Coactivator 3, Ppara Peroxisome Proliferator Activated Receptor Alpha, Ppard Peroxisome Proliferator Activated Receptor Delta, Pparg Peroxisome Proliferator Activated Receptor Gamma, Pten Phosphatase And Tensin Homolog, Plin3 Perilipin 3. **Table S3**. Expression level data of genes involved in lipid metabolism, trafficking and inflammation in the four experimental groups of the in vitro experiments (n=5 for each group). Data are presented as mean±SD and compared by the two-way ANOVA and corrected for multiple testing by the two stage step-up Benjamini, Krieger and Yekutieli false discovery rate method at an alpha level of 0.05. Cd36 Cluster of differentiation 36, Il1b Interleukin-1β, Il6 Interleukin-6, Lpl Lipoprotein Lipase, Ppara Peroxisome Proliferator Activated Receptor Alpha, Pparg Peroxisome Proliferator Activated Receptor Gamma, Plin3 Perilipin 3, Tnfa Tumour Necrosis Factor-α. **Table S4**. Anthropometric, clinical and biochemical characteristics of the study population. Data are presented as median [IQR] and compared by the Mann-Whitney U test and the Fischer’s exact test for categorical variables. ACE-i: angiotensin-converting enzyme inhibitors ARBs angiotensin receptor blockers, BMI body mass index, EF ejection fraction, FS fractional shortening HDL-C high-density lipoprotein cholesterol, LDL-C low-density lipoprotein cholesterol, LVEDD left ventricular end-diastolic diameter, LVESD left ventricular end-systolic diameter, LVM: left ventricular mass, T2D type 2 diabetes.

## Data Availability

The datasets used and/or analysed during the current study are available from the corresponding author upon request.

## Abbreviations

AET: Aortic ejection time
EDV: End-diastolic volume
EF: Ejection fraction
ESV: End-systolic volume
FA: Fatty acid
FS: Fractional shortening
HFpEF: Heart failure with preserved ejection fraction
HG: High glucose
IVCT: Isovolumic contraction time
IVRT: Isovolumic relaxation time
LCT: Long-chain triacylglycerols
LV: Left ventricle
LVIDd: Left ventricular end-diastolic internal diameter
LVIDs: Left ventricular end-systolic internal diameter
MCM: Metabolic cardiomyopathy
MDA: Malondialdehyde
MLCT: Medium and long-chain triacylglycerols
MMC: MTBE: chloroform
MPI: Myocardial performance index
NG: Normal glucose
PBS: Phosphate-buffered saline
rSirt1: Recombinant Sirt1
SFA: Saturated fatty acid
SGLT-2i: SGLT-2 inhibitors
Sirt1: Mammalian silent information regulator 1
STRIN: Search Tool for the Retrieval of Interacting Genes/Proteins
T2D: Type 2 diabetes
TAG: Triacylglycerols
TDI: Tissue Doppler Imaging
VLCT: Very long-chain triacylglycerols

## References

[1] Chen Z, Jin ZX, Cai J, Li R, Deng KQ, Ji YX (2022). Energy substrate metabolism and oxidative stress in metabolic cardiomyopathy. J Mol Med (Berl).

[2] Schiattarella GG, Altamirano F, Tong D, French KM, Villalobos E, Kim SY (2019). Nitrosative stress drives heart failure with preserved ejection fraction. Nature.

[3] Costantino S, Paneni F, Cosentino F (2016). Ageing, metabolism and cardiovascular disease. J Physiol.

[4] Chew NWS, Ng CH, Tan DJH, Kong G, Lin C, Chin YH (2023). The global burden of metabolic disease: data from 2000 to 2019. Cell Metab.

[5] Nishida K, Otsu K (2017). Inflammation and metabolic cardiomyopathy. Cardiovasc Res.

[6] McHugh K, DeVore AD, Wu J, Matsouaka RA, Fonarow GC, Heidenreich PA (2019). Heart failure with preserved ejection fraction and diabetes: JACC state-of-the-art review. J Am Coll Cardiol.

[7] Suffee N, Baptista E, Piquereau J, Ponnaiah M, Doisne N, Ichou F (2022). Impacts of a high-fat diet on the metabolic profile and the phenotype of atrial myocardium in mice. Cardiovasc Res.

[8] Costantino S, Akhmedov A, Melina G, Mohammed SA, Othman A, Ambrosini S (2019). Obesity-induced activation of JunD promotes myocardial lipid accumulation and metabolic cardiomyopathy. Eur Heart J.

[9] Karbasforooshan H, Karimi G (2017). The role of SIRT1 in diabetic cardiomyopathy. Biomed Pharmacother.

[10] Jia G, Hill MA, Sowers JR (2018). Diabetic cardiomyopathy. Circ Res.

[11] Tan Y, Zhang Z, Zheng C, Wintergerst KA, Keller BB, Cai L (2020). Mechanisms of diabetic cardiomyopathy and potential therapeutic strategies: preclinical and clinical evidence. Nat Rev Cardiol.

[12] Kane AE, Sinclair DA (2018). Sirtuins and NAD + in the development and treatment of metabolic and cardiovascular diseases. Circ Res.

[13] Mengozzi A, Costantino S, Paneni F, Duranti E, Nannipieri M, Mancini R (2022). Targeting SIRT1 rescues age- and obesity-induced microvascular dysfunction in ex-vivo human vessels. Circ Res.

[14] Winnik S, Auwerx J, Sinclair DA, Matter CM (2015). Protective effects of sirtuins in cardiovascular diseases: from bench to bedside. Eur Heart J.

[15] Liang F, Kume S, Koya D (2009). SIRT1 and insulin resistance. Nat Rev Endocrinol.

[16] Li Y, Wong K, Giles A, Jiang J, Lee JW, Adams AC (2014). Hepatic SIRT1 attenuates hepatic steatosis and controls energy balance in mice by inducing fibroblast growth factor 21. Gastroenterology.

[17] Yamamoto T, Sano M (2022). Deranged myocardial fatty acid metabolism in heart failure. Int J Mol Sci.

[18] Paulus WJ, Tschöpe C (2013). A novel paradigm for heart failure with preserved ejection fraction: comorbidities drive myocardial dysfunction and remodeling through coronary microvascular endothelial inflammation. J Am Coll Cardiol.

[19] Packer M (2020). Cardioprotective effects of sirtuin-1 and its downstream effectors: potential role in mediating the heart failure benefits of SGLT2 (Sodium-Glucose Cotransporter 2) inhibitors. Circ Heart Fail.

[20] Wang YJ, Paneni F, Stein S, Matter CM (2021). Modulating sirtuin biology and nicotinamide adenine diphosphate metabolism in cardiovascular disease-from bench to bedside. Front Physiol.

[21] Jalgaonkar MP, Parmar UM, Kulkarni YA, Oza MJ (2022). SIRT1-FOXOs activity regulates diabetic complications. Pharmacol Res.

[22] Paneni F, Costantino S, Castello L, Battista R, Capretti G, Chiandotto S (2015). Targeting prolyl-isomerase Pin1 prevents mitochondrial oxidative stress and vascular dysfunction: insights in patients with diabetes. Eur Heart J.

[23] Pellegrino RM, Di Veroli A, Valeri A, Goracci L, Cruciani G (2014). LC/MS lipid profiling from human serum: a new method for global lipid extraction. Anal Bioanal Chem.

[24] Narvaez-Rivas M, Zhang Q (2016). Comprehensive untargeted lipidomic analysis using core-shell C30 particle column and high field orbitrap mass spectrometer. J Chromatogr A.

[25] Classification and Diagnosis of Diabetes (2018). Standards of medical care in diabetes-2018. Diabetes Care.

[26] Yadav SK, Kambis TN, Kar S, Park SY, Mishra PK (2020). MMP9 mediates acute hyperglycemia-induced human cardiac stem cell death by upregulating apoptosis and pyroptosis in vitro. Cell Death Dis.

[27] Wang W, Gu H, Li W, Lin Y, Yao X, Luo W (2021). SRC-3 knockout attenuates myocardial injury induced by chronic intermittent hypoxia in mice. Oxid Med Cell Longev.

[28] Qiang L, Wang L, Kon N, Zhao W, Lee S, Zhang Y (2012). Brown remodeling of white adipose tissue by SirT1-dependent deacetylation of Pparγ. Cell.

[29] Wenzl FA, Ambrosini S, Mohammed SA, Kraler S, Lüscher TF, Costantino S (2021). Inflammation in metabolic cardiomyopathy. Front Cardiovasc Med.

[30] Peterson LR, Gropler RJ (2020). Metabolic and molecular imaging of the diabetic cardiomyopathy. Circ Res.

[31] Schiattarella GG, Rodolico D, Hill JA (2020). Metabolic inflammation in heart failure with preserved ejection fraction. Cardiovasc Res.

[32] Borlaug BA, Sharma K, Shah SJ, Ho JE (2023). Heart failure with preserved ejection fraction: JACC scientific statement. J Am Coll Cardiol.

[33] Gorski PA, Jang SP, Jeong D, Lee A, Lee P, Oh JG (2019). Role of SIRT1 in modulating acetylation of the sarco-endoplasmic reticulum Ca(2+)-ATPase in heart failure. Circ Res.

[34] Planavila A, Dominguez E, Navarro M, Vinciguerra M, Iglesias R, Giralt M (2012). Dilated cardiomyopathy and mitochondrial dysfunction in Sirt1-deficient mice: a role for Sirt1-Mef2 in adult heart. J Mol Cell Cardiol.

[35] Vilella R, Izzo S, Naponelli V, Savi M, Bocchi L, Dallabona C (2022). In vivo treatment with a standardized green tea extract restores cardiomyocyte contractility in diabetic rats by improving mitochondrial function through SIRT1 activation. Pharmaceuticals (Basel).

[36] Ren BC, Zhang YF, Liu SS, Cheng XJ, Yang X, Cui XG (2020). Curcumin alleviates oxidative stress and inhibits apoptosis in diabetic cardiomyopathy via Sirt1-Foxo1 and PI3K-Akt signalling pathways. J Cell Mol Med.

[37] Huang Q, Su H, Qi B, Wang Y, Yan K, Wang X (2021). A SIRT1 activator, ginsenoside Rc, promotes energy metabolism in cardiomyocytes and neurons. J Am Chem Soc.

[38] Waldman M, Cohen K, Yadin D, Nudelman V, Gorfil D, Laniado-Schwartzman M (2018). Regulation of diabetic cardiomyopathy by caloric restriction is mediated by intracellular signaling pathways involving 'SIRT1 and PGC-1α'. Cardiovasc Diabetol.

[39] Hou X, Rooklin D, Fang H, Zhang Y (2016). Resveratrol serves as a protein-substrate interaction stabilizer in human SIRT1 activation. Sci Rep.

[40] Sulaiman M, Matta MJ, Sunderesan NR, Gupta MP, Periasamy M, Gupta M (2010). Resveratrol, an activator of SIRT1, upregulates sarcoplasmic calcium ATPase and improves cardiac function in diabetic cardiomyopathy. Am J Physiol Heart Circ Physiol.

[41] Gu XS, Wang ZB, Ye Z, Lei JP, Li L, Su DF (2014). Resveratrol, an activator of SIRT1, upregulates AMPK and improves cardiac function in heart failure. Genet Mol Res.

[42] Ren J, Wu NN, Wang S, Sowers JR, Zhang Y (2021). Obesity cardiomyopathy: evidence, mechanisms, and therapeutic implications. Physiol Rev.

[43] Björnson E, Östlund Y, Ståhlman M, Adiels M, Omerovic E, Jeppsson A (2020). Lipid profiling of human diabetic myocardium reveals differences in triglyceride fatty acyl chain length and degree of saturation. Int J Cardiol.

[44] Hammer S, Snel M, Lamb HJ, Jazet IM, van der Meer RW, Pijl H (2008). Prolonged caloric restriction in obese patients with type 2 diabetes mellitus decreases myocardial triglyceride content and improves myocardial function. J Am Coll Cardiol.

[45] Mason RP, Libby P, Bhatt DL (2020). Emerging mechanisms of cardiovascular protection for the omega-3 fatty acid eicosapentaenoic acid. Arterioscler Thromb Vasc Biol.

[46] Shi Y, Li H, Wu T, Wang Q, Zhu Q, Guan X (2022). Docosahexaenoic acid-enhanced autophagic flux improves cardiac dysfunction after myocardial infarction by targeting the AMPK/mTOR signaling pathway. Oxid Med Cell Longev.

[47] Tucci S, Behringer S, Spiekerkoetter U (2015). De novo fatty acid biosynthesis and elongation in very long-chain acyl-CoA dehydrogenase-deficient mice supplemented with odd or even medium-chain fatty acids. FEBS J.

[48] Wang L, Cai Y, Jian L, Cheung CW, Zhang L, Xia Z (2021). Impact of peroxisome proliferator-activated receptor-α on diabetic cardiomyopathy. Cardiovasc Diabetol.

[49] Li H, Fan J, Zhao Y, Zhang X, Dai B, Zhan J (2019). Nuclear miR-320 mediates diabetes-induced cardiac dysfunction by activating transcription of fatty acid metabolic genes to cause lipotoxicity in the heart. Circ Res.

[50] Li Y, Yang M, Tan J, Shen C, Deng S, Fu X (2022). Targeting ACSL1 promotes cardiomyocyte proliferation and cardiac regeneration. Life Sci.

[51] Tsushima K, Bugger H, Wende AR, Soto J, Jenson GA, Tor AR (2018). Mitochondrial reactive oxygen species in lipotoxic hearts induce post-translational modifications of AKAP121, DRP1, and OPA1 that promote mitochondrial fission. Circ Res.

[52] ElSayed NA, Aleppo G, Aroda VR, Bannuru RR, Brown FM, Bruemmer D (2023). 2. Classification and diagnosis of diabetes: standards of care in diabetes-2023. Diabet Care.

[53] Zhao Y, Zhang J, Zheng Y, Zhang Y, Zhang XJ, Wang H (2021). NAD(+) improves cognitive function and reduces neuroinflammation by ameliorating mitochondrial damage and decreasing ROS production in chronic cerebral hypoperfusion models through Sirt1/PGC-1α pathway. J Neuroinflammation.

[54] Han L, Zhou R, Niu J, McNutt MA, Wang P, Tong T (2010). SIRT1 is regulated by a PPAR{γ}-SIRT1 negative feedback loop associated with senescence. Nucleic Acids Res.

[55] Milne JC, Lambert PD, Schenk S, Carney DP, Smith JJ, Gagne DJ (2007). Small molecule activators of SIRT1 as therapeutics for the treatment of type 2 diabetes. Nature.

[56] Jia W, Bai T, Zeng J, Niu Z, Fan D, Xu X (2021). Combined administration of metformin and atorvastatin attenuates diabetic cardiomyopathy by inhibiting inflammation, apoptosis, and oxidative stress in type 2 diabetic mice. Front Cell Dev Biol.

[57] Gano LB, Donato AJ, Pasha HM, Hearon CM, Sindler AL, Seals DR (2014). The SIRT1 activator SRT1720 reverses vascular endothelial dysfunction, excessive superoxide production, and inflammation with aging in mice. Am J Physiol Heart Circ Physiol.

[58] Yang K, Velagapudi S, Akhmedov A, Kraler S, Lapikova-Bryhinska T, Schmiady MO (2023). Chronic SIRT1 supplementation in diabetic mice improves endothelial function by suppressing oxidative stress. Cardiovasc Res.

