# Supplementary material for: Treatment with recombinant Sirt1 rewires the cardiac lipidome and rescues diabetes-related metabolic cardiomyopathy
Source: Cardiovasc Diabetol. 2023 Nov 13;22:312. doi: 10.1186/s12933-023-02057-2 (PMC10644415; doi:10.1186/s12933-023-02057-2)
Supplement: Supplementary file 1 — Additional file1: Figure S1. Schematic study protocol. At 12 weeks, db/db mice were administered recombinant Sirt1 (rSirt1) by intraperitoneal injection, 0.3 mg/Kg every other day. At 16 weeks, mice cardiac function and structure were evaluated, and then mice were sacrificed and harvested for molecular analyses. rSirt1 recombinant Sirt1. Figure S2. rSirt1 treatment effect on cardiac geometry and physiology. A Left ventricular end-diastolic and end-systolic internal diameters, volumes and mass of the three experimental groups: db/+ mice (black dots and plots; n=11); db/db mice (red dots and plots; n=12); db/db mice after 4-week treatment with rSirt1 (green dots and plots; n=13). B Stroke volume, heart rate and cardiac output of the three experimental groups. Data are presented as violin plots showing median [IQR] and compared by the Kruskal-Wallis test (upper bold bar) with Dunn post hoc test. *p<0.05, **p<0.01. EDV end-diastolic volume, ESV end-systolic volume, LVIDd left ventricular end-diastolic internal diameter, LVIDs left ventricular end-systolic internal diameter; rSirt1: recombinant Sirt1. Table S1. Cardiac lipidomics analysis performed in the three experimental groups (n=6 for each group). Data are presented as median [IQR] and compared by the Kruskal-Wallis test and corrected for multiple testing by the two stage step-up Benjamini, Krieger and Yekutieli false discovery rate method at an alpha level of 0.05. TAG triacylglycerol. Table S2. Expression level data of genes involved in lipid metabolism, trafficking and inflammation in the three experimental groups (n=4 for each group). Data are presented as mean±SD and compared by the two-way ANOVA and corrected for multiple testing by the two stage step-up Benjamini, Krieger and Yekutieli false discovery rate method at an alpha level of 0.05. Acox3 Acyl-CoA Oxidase 3, Acsl1 Acyl-CoA Synthetase Long Chain Family Member 1, Apoa5 Apolipoprotein A5, Apoc3 Apolipoprotein C3, Cd36 Cluster of differentiation 36, Cpt1a [file 12933_2023_2057_MOESM1_ESM.docx]

**ADDITIONAL FILES**

**Treatment with recombinant Sirt1 rewires the cardiac lipidome and rescues diabetes-related metabolic cardiomyopathy**

S. Costantino^1,2^*, A. Mengozzi^1,3,4^*, S. Velagapudi^5^*, S.A. Mohammed^1^, E. Gorica^1^, A. Akhmedov^5^, A. Mongelli^1^, N.R. Pugliese^3^, S. Masi^3^, A. Virdis^3^, A. Hülsmeier^6^, C.M. Matter^1,2^, T. Hornemann^6^, G. Melina^7^, F. Ruschitzka^1,2^, T.F. Luscher^5,8^, F. Paneni^1,2^

^1^Center for Translational and Experimental Cardiology (CTEC), Department of Cardiology, Zurich University Hospital and University of Zurich, Switzerland;

^2^Department of Cardiology, Zurich University Hospital, Switzerland;

^3^Department of Clinical and Experimental Medicine, University of Pisa, Pisa, Italy;

^4^Health Science Interdisciplinary Center, Sant’Anna School of Advanced Studies, Pisa, Italy;

^5^Center for Molecular Cardiology, Zurich, Switzerland;

^6^Institute for Clinical Chemistry, University Hospital and University of Zürich, Zürich, Switzerland;

^7^Department of Clinical and Molecular Medicine, Sapienza University of Rome, Rome, Italy;

^8^Royal Brompton and Harefield Hospitals and Imperial College, London, UK.

*The authors contributed equally to this work.

**Correspondence**:

Prof. Francesco Paneni, MD, PhD, FESC

Center for Translational and Experimental Cardiology (CTEC),

Department of Cardiology, University Hospital Zurich, University of Zurich,

Wagistrasse 12, 8952, Schlieren, Switzerland.

[francesco.paneni@uzh.ch](mailto:francesco.paneni@uzh.ch)

**Additional Figure 1. Schematic study protocol**. At 12 weeks, *db/db* mice were administered recombinant Sirt1 (rSirt1) by intraperitoneal injection, 0.3 mg/Kg every other day. At 16 weeks, mice cardiac function and structure were evaluated, and then mice were sacrificed and harvested for molecular analyses. *rSirt1: recombinant Sirt1.*

**
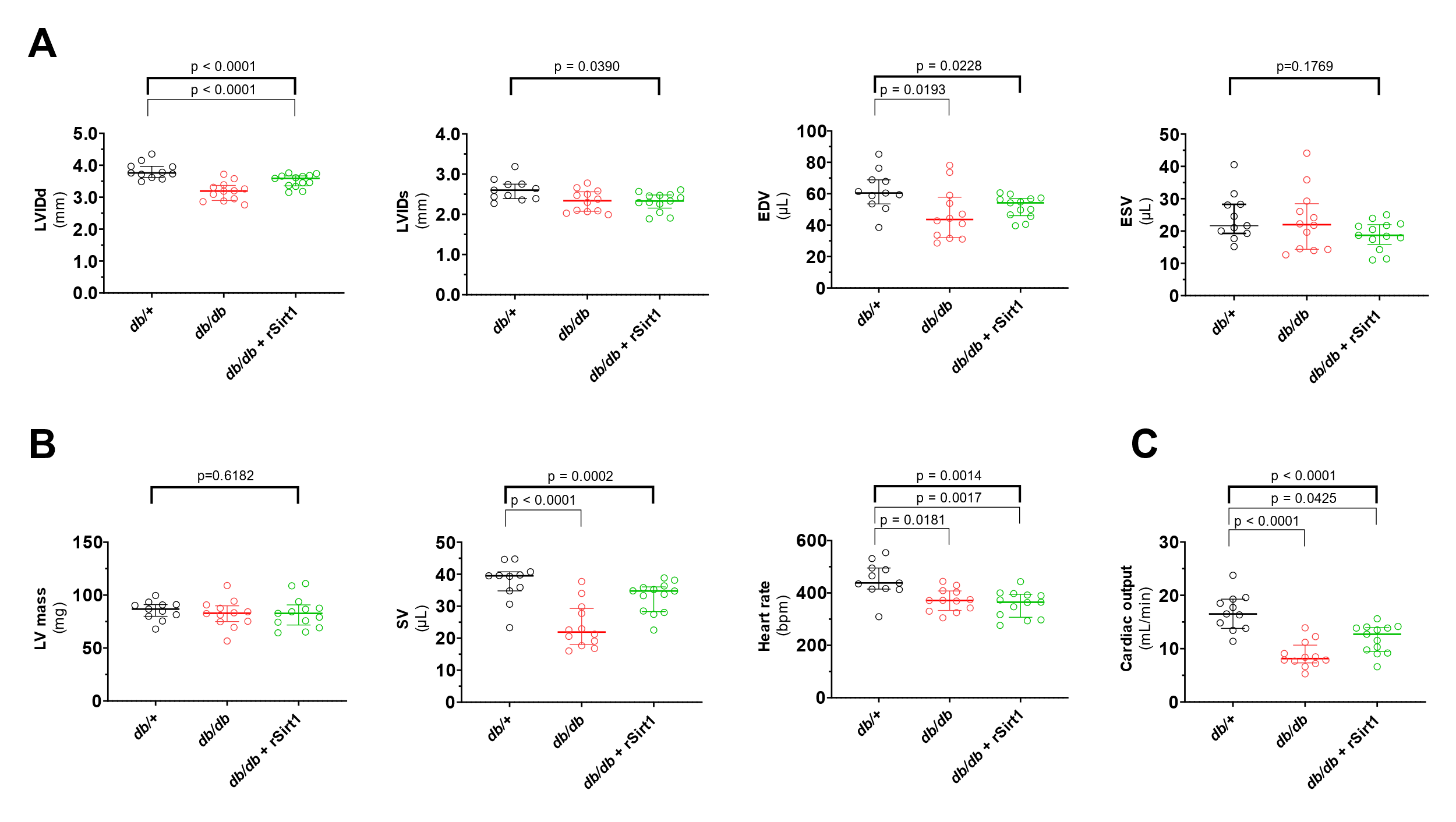
**

**Additional Figure 2. rSirt1 treatment effect on cardiac geometry and physiology.** (**A**) Left ventricular end-diastolic and end-systolic internal diameters, volumes and mass of the three experimental groups: *db/+* mice (black dots and plots; n=11); *db/db* mice (red dots and plots; n=12); *db/db* mice after 4-week treatment with rSirt1 (green dots and plots; n=13). (**B**) Stroke volume, heart rate and cardiac output of the three experimental groups. Data are presented as violin plots showing median [IQR] and compared by the Kruskal-Wallis test (upper bold bar) with Dunn *post hoc* test. *p<0.05, **p<0.01. *EDV: end-diastolic volume; ESV: end-systolic volume; LVIDd: left ventricular end-diastolic internal diameter; LVIDs: left ventricular end-systolic internal diameter; rSirt1: recombinant Sirt1.*

|  | ***db*/+**  **(n=6)** | ***db/db***  **(n=6)** | ***db/db*+rSirt1**  **(n=6)** | **P value**  **(KW)** | **P value**  **(*db*/+ vs *db/db)*** | **P value**  **(*db*/+ vs *db/db*+ rSirt1*)*** | **P value**  **(*db/d* vs *db/db*+ rSirt1*)*** |
| --- | --- | --- | --- | --- | --- | --- | --- |
| TAG (42:1) (12:0/12:0/18:1) | 0.76 [0.50] | 2.20 [0.71] | 2.30 [1.77] | 0.0129 | 0.0151 | 0.0564 | 0.3690 |
| TAG (42:2) (12:0/12:0/18:2) | 0.19 [0.09] | 0.57 [0.49] | 0.82 [1.84] | 0.0756 | 0.1084 | 0.1536 | 0.7550 |
| TAG (44:2) (12:0/14:0/18:2) | 1.32 [0.71] | 4.76 [1.47] | 2.83 [1.29] | <0.0001 | 0.0006 | 0.2060 | 0.0449 |
| TAG (46:2) (12:0/16:0/18:2) | 3.16 [1.97] | 25.77 [8.49] | 13.87 [13.96] | <0.0001 | 0.0010 | 0.0815 | 0.0815 |
| TAG (46:3) (12:0/16:0/18:3) | 0.61 [0.43] | 5.75 [3.33] | 3.00 [3.89] | 0.0001 | 0.0013 | 0.0721 | 0.1030 |
| TAG (48:0) (14:0/16:0/18:0) | 38.55 [18.52] | 398.11 [218.78] | 164.44 [205.80] | <0.0001 | 0.0004 | 0.0231 | 0.1944 |
| TAG (48:2) (14:0/16:0/18:2) | 38.55 [18.52] | 421.65 [153.60] | 164.44 [71.18] | <0.0001 | 0.0002 | 0.0350 | 0.1048 |
| TAG (48:3) | 0.72 [0.33] | 2.59 [1.48] | 1.90 [1.72] | 0.0155 | 0.0122 | 0.1361 | 0.1361 |
| TAG (48:3) (12:0/18:1/18:2) | 2.37 [1.74] | 50.15 [16.09] | 19.55 [12.38] | <0.0001 | 0.0005 | 0.0833 | 0.0564 |
| TAG (50:0) (16:0/16:0/18:0) | 15.60 [5.65] | 185.41 [137.98] | 186.93 [222.05] | 0.0004 | 0.0022 | 0.0031 | 0.2610 |
| TAG (50:3) (14:0/18:1/18:2) | 34.31 [28.09] | 521.77 [369.38] | 226.22 [235.23] | <0.0001 | 0.0004 | 0.0231 | 0.1944 |
| TAG (50:4) (14:0/18:1/18:3) | 3.21 [2.63] | 86.06 [101.33] | 97.83 [113.76] | 0.0010 | 0.0026 | 0.0042 | 0.2468 |
| TAG (50:5) (14:0/18:2/18:3) | 0.20 [0.18] | 21.21 [8.93] | 4.61 [4.44] | <0.0001 | 0.0009 | 0.0656 | 0.0656 |
| TAG (51:1) (16:0/17:0/18:1) | 11.78 [4.63] | 48.26 [26.11] | 47.28 [46.18] | 0.0018 | 0.0037 | 0.0050 | 0.2610 |
| TAG (51:2) (15:0/18:1/18:1) | 13.01 [7.04] | 74.85 [36.42] | 59.63 [57.94] | 0.0015 | 0.0026 | 0.0068 | 0.2060 |
| TAG (51:3) (15:0/18:1/18:2) | 6.41 [4.49] | 52.28 [27.97] | 51.09 [54.99] | 0.0011 | 0.0031 | 0.0031 | 0.3049 |
| TAG (51:4) (15:0/18:1/18:3) | 0.86 [0.57] | 11.20 [11.07] | 14.74 [17.46] | 0.0011 | 0.0031 | 0.0031 | 0.3049 |
| TAG (52:0) (16:0/18:0/18:0) | 3.00 [0.71] | 22.38 [12.10] | 24.76 [29.56] | 0.0005 | 0.0026 | 0.0026 | 0.2901 |
| TAG (52:1) (16:0/16:0/20:1) | 88.55 [16.88] | 600.02 [349.87] | 603.45 [622.58] | 0.0004 | 0.0022 | 0.0031 | 0.2610 |
| TAG (52:4) (16:0/16:0/20:4) | 146.02 [65.40] | 943.21 [682.99] | 1081.48 [1023.82] | 0.0004 | 0.0022 | 0.0031 | 0.2610 |
| TAG (52:4) (16:1/18:1/18:2) | 146.02 [65.40] | 943.21 [682.99] | 1081.48 [1023.82] | 0.0004 | 0.0022 | 0.0031 | 0.2610 |
| TAG (52:5) (14:0/18:1/20:4) | 8.16 [4.75] | 174.27 [232.61] | 231.69 [265.49] | 0.0008 | 0.0022 | 0.0050 | 0.2193 |
| TAG (52:6) (16:0/18:2/18:4) | 0.97 [0.42] | 25.21 [12.65] | 6.84 [5.05] | <0.0001 | 0.0004 | 0.0520 | 0.0520 |
| TAG (53:2) (17:0/18:0/18:2) | 23.44 [10.43] | 90.27 [48.91] | 49.80 [54.71] | 0.0023 | 0.0036 | 0.0584 | 0.2136 |
| TAG (53:3) (17:0/18:1/18:2) | 14.46 [7.56] | 14.46 [7.56] | 45.81 [66.30] | 0.0010 | 0.0025 | 0.0367 | 0.1793 |
| TAG (53:4) (17:0/18:1/18:3) | 3.07 [2.25] | 28.90 [23.50] | 36.53 [40.11] | 0.0021 | 0.0036 | 0.0036 | 0.3199 |
| TAG (54:1) (18:0/18:0/18:1) | 19.83 [15.95] | 113.12 [85.05] | 113.06 [164.77] | 0.0005 | 0.0026 | 0.0026 | 0.2901 |
| TAG (54:2) (16:0/18:1/20:1) | 122.85 [65.39] | 718.72 [381.79] | 684.03 [648.22] | 0.0005 | 0.0026 | 0.0026 | 0.2901 |
| TAG (54:5) (16:0/18:1/20:4) | 37.48 [24.19] | 1029.74 [107.87] | 307.42 [197.77] | <0.0001 | 0.0003 | 0.0462 | 0.0462 |
| TAG (54:7) (16:0/18:2/20:5) | 1.37 [0.59] | 26.38 [38.11] | 37.35 [36.06] | 0.0004 | 0.0022 | 0.0031 | 0.2610 |
| TAG (56:2) (18:0/18:1/20:1) | 38.14 [24.59] | 143.25 [98.16] | 159.04 [201.33] | 0.0050 | 0.0061 | 0.0079 | 0.2610 |
| TAG (56:3) (18:0/18:1/20:2) | 36.00 [34.22] | 361.89 [202.96] | 120.99 [63.87] | <0.0001 | 0.0006 | 0.0585 | 0.0585 |
| TAG (56:5) (16:0/18:1/22:4) | 14.87 [4.09] | 129.70 [172.67] | 134.29 [113.14] | 0.0004 | 0.0022 | 0.0031 | 0.2610 |
| TAC (56:6) (16:0/18:0/22:6) | 27.56 [3.90] | 288.46 [420.40] | 356.56 [334.05] | 0.0005 | 0.0026 | 0.0026 | 0.2901 |
| TAG (56:7) (16:0/18:1/22:6) | 17.14 [4.48] | 253.29 [440.19] | 331.74 [343.85] | 0.0006 | 0.0022 | 0.0022 | 0.3199 |
| TAG (56:8) (16:0/18:2/22:6) | 4.37 [1.89] | 84.23 [176.83] | 109.61 [126.49] | 0.0006 | 0.0022 | 0.0022 | 0.3199 |
| TAG (56:9) (18:1/18:3/20:5) | 0.26 [0.08] | 8.47 [18.10] | 10.46 [14.67] | 0.0006 | 0.0018 | 0.0018 | 0.3500 |
| TAG (58:1) (18:0/18:0/22:1) | 2.01 [1.33] | 8.55 [10.39] | 5.21 [6.71] | 0.0059 | 0.0062 | 0.0877 | 0.1495 |
| TAG (58:3) (18:1/18:1/22:1) | 11.99 [11.49] | 64.93 [69.52] | 86.53 [80.59] | 0.0054 | 0.0068 | 0.0068 | 0.2901 |
| TAG (58:4) (18:1/18:1/22:2) | 4.57 [4.02] | 37.88 [54.06] | 45.52 [41.51] | 0.0031 | 0.0052 | 0.0058 | 0.2754 |
| TAG (58:6) | 0.04 [0.02] | 1.50 [2.22] | 1.58 [1.93] | 0.0022 | 0.0031 | 0.0031 | 0.3500 |
| TAG (58:6) (18:0/18:0/22:6) | 3.56 [0.25] | 61.10 [88.44] | 56.34 [59.60] | 0.0006 | 0.0018 | 0.0018 | 0.3500 |
| TAG (58:8) (18:1/18:1/22:6) | 2.51 [0.75] | 81.22 [169.79] | 90.44 [125.28] | 0.0006 | 0.0022 | 0.0022 | 0.3199 |
| TAG (58:9) (18:1/18:2/22:6) | 1.31 [0.55] | 49.73 [105.64] | 60.32 [84.92] | 0.0006 | 0.0018 | 0.0018 | 0.3500 |
| TAG (58:10) (18:2/18:2/22:6) | 0.57 [0.23] | 15.63 [36.08] | 15.63 [36.08] | 0.0006 | 0.0018 | 0.0018 | 0.3500 |

**Additional Table 1**. Cardiac lipidomics analysis performed in the three experimental groups (n=6 for each group). Data are presented as median [IQR] and compared by the Kruskal-Wallis test and corrected for multiple testing by the two stage step-up Benjamini, Krieger and Yekutieli false discovery rate method at an alpha level of 0.05. *TAG: triacylglycerol.*

|  | ***db*/+**  **(n=4)** | ***db/db***  **(n=4)** | ***db/db*+rSirt1**  **(n=4)** | **P value**  **(ANOVA)** | **P value**  **(*db*/+ vs *db/db)*** | **P value**  **(*db*/+ vs *db/db*+ rSirt1*)*** | **P value**  **(*db/db* vs *db/db*+ rSirt1*)*** |
| --- | --- | --- | --- | --- | --- | --- | --- |
| *Acox3* | 1±0.32 | 5.66±0.14 | 1.15±0.33 | <0.0001 | <0.0001 | 0.1920 | <0.0001 |
| *Acsl1* | 1±0.68 | 0.35±0.52 | 0.83±0.36 | <0.0001 | 0.0234 | 0.3596 | 0.0603 |
| *Apoa5* | 1±0.50 | 2.41±0.38 | 0.84±0.46 | <0.0001 | <0.0001 | 0.1838 | <0.0001 |
| *Apoc3* | 1±0.11 | 2.42±0.24 | 0.77±0.54 | <0.0001 | <0.0001 | 0.1285 | <0.0001 |
| *Cd36* | 1±1.02 | 7.17±0.16 | 1.15±0.35 | <0.0001 | <0.0001 | 0.1909 | <0.0001 |
| *Cpt1a* | 1±0.24 | 3.40±0.47 | 1.19±0.46 | <0.0001 | <0.0001 | 0.1561 | <0.0001 |
| *Cpt1b* | 1±0.06 | 2.49±0.07 | 1.17±0.33 | <0.0001 | <0.0001 | 0.1797 | <0.0001 |
| *Fabp1* | 1±0.61 | 2.43±0.37 | 1.06±0.37 | <0.0001 | <0.0001 | 0.2840 | <0.0001 |
| *Fabp2* | 1±0.45 | 7.11±0.20 | 2.27±0.23 | <0.0001 | <0.0001 | <0.0001 | <0.0001 |
| *Fads2* | 1±0.06 | 3.64±0.07 | 1.89±0.23 | <0.0001 | <0.0001 | 0.0007 | <0.0001 |
| *Lpl* | 1±0.33 | 5.24±0.16 | 1.39±0.08 | <0.0001 | <0.0001 | 0.0443 | <0.0001 |
| *Il1b* | 1±0.45 | 2.15±0.21 | 1.09±0.33 | 0.0002 | 0.0002 | 0.2273 | 0.0002 |
| *Il6* | 1±0.30 | 2.58±0.69 | 1.27±0.23 | 0.0002 | <0.0001 | 0.1427 | 0.0001 |
| *Mmp9* | 1±0.12 | 8.37±0.45 | 3.28±0.91 | <0.0001 | <0.0001 | <0.0001 | <0.0001 |
| *Ncoa3* | 1±0.07 | 3.45±0.23 | 0.83±0.28 | <0.0001 | <0.0001 | 0.1770 | <0.0001 |
| *Ppara* | 1±0.06 | 4.36±0.07 | 1.06±0.38 | <0.0001 | <0.0001 | 0.2840 | <0.0001 |
| *Ppard* | 1±0.17 | 2.29±0.07 | 1.21±0.10 | <0.0001 | <0.0001 | 0.1424 | <0.0001 |
| *Pparg* | 1±0.13 | 5.05±0.05 | 1.15±0.37 | <0.0001 | <0.0001 | 0.1955 | <0.0001 |
| *Pten* | 1±0.26 | 3.23±0.39 | 1.51±0.18 | <0.0001 | <0.0001 | 0.0167 | <0.0001 |
| *Plin3* | 1±0.71 | 3.87±0.23 | 1.32±0.37 | <0.0001 | <0.0001 | 0.0731 | <0.0001 |
| *Tnfa* | 1±0.30 | 1.89±0.38 | 1.13±0.13 | 0.0072 | 0.0038 | 0.2033 | 0.0043 |

**Additional Table 2**. Expression level data of genes involved in lipid metabolism, trafficking and inflammation in the three experimental groups (n=4 for each group). Data are presented as mean±SD and compared by the two-way ANOVA and corrected for multiple testing by the two stage step-up Benjamini, Krieger and Yekutieli false discovery rate method at an alpha level of 0.05. *Acox3: Acyl-CoA Oxidase 3; Acsl1: Acyl-CoA Synthetase Long Chain Family Member 1, Apoa5: Apolipoprotein A5; Apoc3: Apolipoprotein C3; Cd36: Cluster of differentiation 36; Cpt1a: Carnitine Palmitoyltransferase 1A; Cpt1b: Carnitine Palmitoyltransferase 1B; Fabp1: Fatty Acid Binding Protein 1; Fabp2: Fatty Acid Binding Protein 2; Fads2: Fatty Acid Desaturase 2; Lpl: Lipoprotein Lipase; Mmp9: Matrix Metallopeptidase 9; Ncoa3: Nuclear Receptor Coactivator 3; Ppara: Peroxisome Proliferator Activated Receptor Alpha; Ppard: Peroxisome Proliferator Activated Receptor Delta; Pparg: Peroxisome Proliferator Activated Receptor Gamma; Pten: Phosphatase And Tensin Homolog; Plin3: Perilipin 3.*

|  | **NG**  **(n=5)** | **HG**  **(n=5)** | **HG+rSirt1**  **(n=5)** | **HG+vehicle**  **(n=5)** | **P value**  **(ANOVA)** | **P value**  **(NG**  **vs**  **HG)** | **P value**  **(NG**  **vs HG+rSirt1)** | **P value**  **(NG**  **vs HG+vehicle)** | **P value**  **(HG**  **vs HG+rSirt1)** | **P value**  **(HG**  **vs HG+vehicle)** | **P value**  **(HG+rSirt1 vs HG+vehicle)** |
| --- | --- | --- | --- | --- | --- | --- | --- | --- | --- | --- | --- |
| *Cd36* | 1±0.35 | 2.07±0.66 | 1.00±0.33 | 1.89±0.31 | <0.0001 | <0.0001 | 0.3459 | 0.0002 | <0.0001 | 0.1908 | 0.0002 |
| *Il1b* | 1±0.44 | 2.01±0.53 | 1.06±0.27 | 1.93±0.28 | <0.0001 | <0.0001 | 0.2789 | 0.0001 | 0.0001 | 0.2789 | 0.0003 |
| *Il6* | 1±0.31 | 2.15±0.75 | 0.84±0.26 | 2.25±0.64 | <0.0001 | <0.0001 | 0.2120 | <0.0001 | <0.0001 | 0.2368 | <0.0001 |
| *Lpl* | 1±0.29 | 1.92±0.40 | 1.08±0.43 | 1.92±0.38 | <0.0001 | 0.0002 | 0.3094 | 0.0002 | 0.0004 | 0.3467 | 0.0004 |
| *Ppara* | 1±0.25 | 1.64±0.20 | 0.95±0.19 | 1.97±0.21 | <0.0001 | 0.0050 | 0.2936 | 0.0001 | 0.0037 | 0.0731 | 0.0001 |
| *Pparg* | 1±0.30 | 1.79±0.17 | 1.17±0.32 | 1.72±0.12 | <0.0001 | 0.0029 | 0.2055 | 0.0036 | 0.0078 | 0.2721 | 0.0123 |
| *Plin3* | 1±0.23 | 1.86±0.46 | 1.04±0.16 | 1.85±0.43 | <0.0001 | 0.0006 | 0.3282 | 0.0006 | 0.0006 | 0.3282 | 0.0006 |
| *Tnfa* | 1±0.44 | 1.85±0.43 | 0.91±0.28 | 1.88±0.50 | <0.0001 | 0.0003 | 0.2997 | 0.0003 | 0.0002 | 0.3154 | 0.0002 |

**Additional Table 3**. Expression level data of genes involved in lipid metabolism, trafficking and inflammation in the four experimental groups of the in vitro experiments (n=5 for each group). Data are presented as mean±SD and compared by the two-way ANOVA and corrected for multiple testing by the two stage step-up Benjamini, Krieger and Yekutieli false discovery rate method at an alpha level of 0.05. *Cd36: Cluster of differentiation 36; Il1b: Interleukin-1β; Il6: Interleukin-6; Lpl: Lipoprotein Lipase; Ppara: Peroxisome Proliferator Activated Receptor Alpha; Pparg: Peroxisome Proliferator Activated Receptor Gamma; Plin3: Perilipin 3; Tnfa: Tumour Necrosis Factor-α.*

|  | **Controls**  **(n=9)** | **T2D**  **(n=9)** | **P value** |
| --- | --- | --- | --- |
| Demographics | | | |
| Age (years) | 54 [12] | 59 [13] | 0.198 |
| Gender (F:M) | (8:1) | (6:3) | 0.576 |
| BMI (kg*m^-2^) | 29 [5] | 28 [4] | 0.656 |
| Systolic blood pressure (mmHg) | 125 [10] | 130 [10] | 0.135 |
| Diastolic blood pressure (mmHg) | 80 [20] | 80 [10] | 0.887 |
| Laboratory values | | | |
| Fasting plasma glucose (mg/dL) | 93 [16] | 131 [41] | 0.004 |
| HbA1c (mmol/mol) | 42.2 [0.1] | 52.0 [14.2] | 0.005 |
| Plasma triglycerides (mg/dL) | 142 [59] | 70 [40] | 0.093 |
| HDL-C (mg/dL) | 40 [15] | 35 [14] | 0.656 |
| LDL-C (mg/dL) | 129 [57] | 102 [37] | 0.331 |
| Total cholesterol (mg/dL) | 193 [60] | 148 [61] | 0.250 |
| Medications | | | |
| Statins (%) | 11 | 22 | 1.000 |
| ACE-i/ARBs (%) | 22 | 33 | 0.153 |
| Beta-blockers (%) | 33 | 67 | 0.347 |
| Diuretics (%) | 0 | 33 | 0.206 |
| Oral antidiabetic agents (%) | 0 | 78 | 0.002 |
| Insulin (%) | 0 | 44 | 0.082 |
| Cardiac Function | | | |
| LVEDD (mm) | 60.0 [15.0] | 51.0 [5.8] | 0.233 |
| LVESD (mm) | 36.0 [16.0] | 32.0 [10.0] | 0.376 |
| EF (%) | 58 [12] | 56 [20] | 0.723 |
| FS (%) | 30 [14] | 38 [11] | 0.671 |
| E/A | 1.3 [0.4] | 1.1 [0.6] | 0.079 |
| LVM (g) | 220 [59] | 172 [99] | 0.546 |

**Additional Table 4**. Anthropometric, clinical and biochemical characteristics of the study population. Data are presented as median [IQR] and compared by the Mann-Whitney U test and the Fischer’s exact test for categorical variables. *ACE-i: angiotensin-converting enzyme inhibitors; ARBs: angiotensin receptor blockers; BMI: body mass index; EF: ejection fraction; FS: fractional shortening; HDL-C: high-density lipoprotein cholesterol; LDL-C: low-density lipoprotein cholesterol; LVEDD: left ventricular end-diastolic diameter; LVESD: left ventricular end-systolic diameter; LVM: left ventricular mass; T2D: type 2 diabetes.*
